# Altered protein secretions during interactions between adipose tissue- or bone marrow-derived stromal cells and inflammatory cells

**DOI:** 10.1186/s13287-015-0052-y

**Published:** 2015-04-16

**Authors:** Hidemi Hattori, Masayuki Ishihara

**Affiliations:** Division of Biomedical Engineering, Research Institute, National Defense Medical College, 3-2 Namiki, Tokorozawa, Saitama 359-8513 Japan

## Abstract

**Introduction:**

Paracrine effects can be exploited in cell-based therapies that secrete factors, such as chemokines and cytokines, and can recruit inflammatory cells to transplants. In this study, mouse adipose tissue-derived stromal cells (ASCs) and bone marrow-derived stromal cells (ST2 cells) were used to examine changes in paracrine interactions with inflammation cells.

**Methods:**

Green fluorescent protein positive (GFP^+^) bone marrow cells (BMCs) were injected into an irradiated mouse via the femoral vein, and ASCs and ST2 cells were transplanted intradermally. Subsequently, an *in vivo* imaging system was used to observe behaviors of GFP^+^ BMCs. To detect bone marrow-derived inflammatory cells which migrated to the ASC and ST2 cell transplantation area, the sections were immunostained using antibodies against Gr1, CD11c, and F4/80, and secretory proteins were detected in culture medium using enzyme-linked immunosorbent assay.

**Results:**

Many bone marrow-derived inflammatory cells migrated to ASC and ST2 cell transplantation sites. Among these, neutrophils were detected during the early period and macrophages were predominantly detected at a later point in time. Many chemokines, cytokines, growth factors, matrix metalloproteinases (MMPs), and tissue inhibitors of metalloproteinases (TIMPs) were secreted in abundance from ASCs, and the secretion increased by co-culturing with inflammatory cells, except for secretions of insulin-like growth factor-1, MMP-9 and MMP-13. Although secretions from ST2 cells were less than those from ASCs, co-culture with inflammatory cells increased these secretions to levels similar to those of ASCs. However, unlike ASCs, the ST2 cells did not secrete angiostatin, MMP-2, or MMP-3. Finally, ASCs secreted not only proinflammatory cytokines, angiogenic factors and MMPs but also anti-inflammatory cytokines, anti-angiogenesis factors, and TIMPs.

**Conclusions:**

The effects of cell-based therapies using ASCs and ST2 cells are depended on paracrine effects that are mediated by chemokines, cytokines, growth factors, MMPs, and TIMPs, which comprise responses to interactions between transplanted cells and inflammatory cells. Moreover, paracrine effects of transplanted cells are influenced by inflammatory cells, and are moderated by a balance of secreted inhibitors.

**Electronic supplementary material:**

The online version of this article (doi:10.1186/s13287-015-0052-y) contains supplementary material, which is available to authorized users.

## Introduction

Numerous previous studies report the effects of cell-derived paracrine factors, and adipose tissue- and bone marrow-derived cells have been used as sources for clinical treatments and trials [1]. However, prognostic assessments vary [2] and expectations of treatment effects are often not met [3,4]. Thus, in addition to advantageous effects, the disadvantageous side effects of these cell types should be investigated prior to therapeutic use.

Wound healing is a complex and dynamic process that is influenced by many factors, including cytokines, growth factors, and chemokines [5], and is characterized by precisely programmed phases of coagulation, inflammation, proliferation, and remodeling [6]. Although inflammation is an indispensable biological process, excessive inflammation causes tissue damage and disrupts engraftment of transplanted cells. Previously, we showed that inflammation was involved in cell transplantation [7]. After subcutaneous transplantation of adipose tissue-derived stromal cells (ASCs), bone marrow-derived inflammatory cells, including granulocytes, neutrophils, monocytes, and macrophages, migrated toward ASC transplants; their interactions with ASCs led to secretion of various inflammatory and angiogenic factors and induced extensive angiogenesis [7].

Currently, paracrine effects have been found to play important roles in tissue regeneration and repair [8] and are under consideration as mechanisms that can be exploited in cell-based therapies. In particular, mesenchymal stem/stromal cells from bone marrow and adipose tissues secrete a wide variety of cytokines and growth factors that may be involved in tissue repair [8-10]. Although the paracrine effect is important for tissue regeneration, it remains unclear whether it can be exploited in the treatment of all diseases. Moreover, although transplantation of ASCs induces inflammation, it is unclear whether this may be effective against autoimmune and chronic inflammatory diseases, such as rheumatoid arthritis, osteoarthritis, and diabetic ulcers, and subsequent excess inflammation may have deleterious effects.

In the present study, we initially determined the specificity of paracrine mechanisms for ASC transplants. Subsequently, we compared the effects of ASCs and bone marrow-derived stromal cells (ST2 cells) during the inflammatory stage of wound healing, and investigated their competence as sources for cell-based therapies.

## Methods

### Preparation of cells

The Ethics Committee of Animal Care and Experimentation of the National Defense Medical College (Saitama, Japan) approved the protocol for animal treatment and the intended procedures of the present study. ASCs were prepared from inguinal adipose tissues of 8-week-old male C57BL/6 mice (Japan SLC, Shizuoka, Japan). Adipose tissues were extensively washed in Dulbecco’s modified Eagle’s medium (DMEM) and were digested for 2 hours at 37°C with 0.1% collagenase type I. The samples were resuspended in DMEM, passed through a 40-μm nylon mesh, and then centrifuged at 1,600 rpm for 10 minutes. Cell pellets were resuspended in lysis buffer for a few minutes, DMEM was added, and cells were resuspended and centrifuged at 1,600 rpm for 10 minutes. The cells were then resuspended in DMEM containing 5% heat-inactivated fetal bovine serum and antibiotics (control medium) and were plated. ASCs were subcultured every 4 days and were used in experiments after two passages. Bone marrow-derived stromal cells (ST2 cells) were purchased from Riken BioResource Center (Ibaraki, Japan).

### Flow cytometric analyses

At least 2 × 10^5^ cells were suspended in 30 μL phosphate-buffered saline (PBS) containing 1% fetal bovine serum and incubated for 20 minutes in the presence of phycoerythrin-labeled antibodies specific for CD29, Sca-1, CD90.2, CD105, CD73, CD117, CD34, CD133, CD45, Gr-1, CD11c, and F4/80, or with fluorescein isothiocyanate-labeled antibodies against CD31 (Beckman Coulter, Fullerton, CA, USA). Nonspecific fluorescence was determined using immunoglobulin G for each antibody isotype (eBioscience, San Diego, CA, USA).

### Migration of green fluorescent protein positive bone marrow cells to tissue surrounding a wound, and into the ASC and ST2 cell transplantation area

*In vivo* imaging was performed using fluorescent labeled cells and an OV110 system (Olympus, Tokyo, Japan) as previously described [7]. Green fluorescent protein-positive (GFP^+^) bone marrow cells (BMCs) were extracted by flushing femurs and tibias of male C57BL/6-Tg (CAG-eGFP) mice aged 8 to 13 weeks (Japan SLC). C57BL/6 mice were then irradiated with 6 Gy and hairs were removed from an ear using depilatory cream. On the following day, GFP^+^ BMCs (2.0 × 10^7^ cells/mouse) were injected into tail veins. One day later, a 2.0-mm hole was made in the center of both ears of each mouse using a metal ear punch (Natsume Seisakusho Co. Ltd, Tokyo, Japan) to produce a regenerative dermal tissue model. ASCs and ST2 cells were labeled with PKH26 (Sigma–Aldrich, St. Louis, MO, USA) and suspended in PBS. Subsequently, PKH26-labeled ASCs and ST2 cells (2.0 × 10^6^ cells/5 μL) were transplanted into mouse ears 1 day after injections of GFP^+^ BMCs as described above, and 5 μL normal saline solution of Japanese Pharmacopoeia was injected as a negative control. Transplanted PKH26-labeled cells and migrating GFP^+^ BMCs were then observed using an OV110 system. During observation, anesthesia was induced by inhalation of 1.5% isoflurane in oxygen.

### Immunostaining

Mice were anesthetized with pentobarbital (Kyoritsu Seiyaku Co., Tokyo, Japan) and were perfusion-fixed with 4% paraformaldehyde. Ears were then removed and cryoembedded in optimum cutting temperature compound (Sakura Finetek, Tokyo, Japan), and 10-μm sections were cut using a cryostat. Cryosections were refixed in 4% paraformaldehyde for 15 minutes and were incubated for 1 hour at room temperature in PBS containing skin milk. After blocking, cryosections were incubated for 1 hour with primary biotinylated antibodies specific for Gr-1, CD11c, and F4/80 (1:50 dilutions; eBioscience). Cryosections were then incubated for 30 minutes with allophycocyanin-conjugated streptavidin (1:100 dilution; eBioscience) and nuclei were stained using Hoechst 33258 (Dojindo Laboratories, Kumamoto, Japan).

### Isolation of inflammatory cells

Male C57BL/6 mice (Japan SLC; 8 to 13 weeks old) received intraperitoneal transplantations of 2 mL 12% sodium casein in saline. Gr-1^+^ cells in peritoneal cavities were then collected by laparotomy 1 day after induction, and CD11c^+^ cells and F4/80^+^ cells were collected after 7 days. Cell populations were separated to high purity (>97%) using a magnetic cell sorting method (Miltenyi Biotec, Bergisch Gladbach, Germany) with antibodies against Gr-1, CD11c, and F4/80.

### Detection of secreted factors

ASCs and ST2 cells were plated at 5.0 × 10^4^ cells/well in 48-well culture plates (Sumitomo Bakelite Co., Tokyo, Japan) and were allowed to attach overnight. Subsequently, supernatants were replaced and equal numbers of Gr-1^+^, CD11c^+^, and F4/80^+^ cells were added. After 2 days of culture, the concentrations of secreted proteins were measured from the supernatant using Bio-Plex assays (Bio-Rad, Hercules, CA, USA) for the macrophage inflammatory proteins (MIP)-1α, MIP-1β, and MIP-2, keratinocyte-derived chemokine (KC), granulocyte-colony stimulating factor (G-CSF), monokine induced by interferon γ (MIG), interleukin (IL)-6, and vascular endothelial growth factor (VEGF). Enzyme-linked immunosorbent assays were used to detect monocyte chemotactic protein (MCP)-1 (Thermo Fisher, Boston, MA, USA), hepatocyte growth factor (HGF), insulin-like growth factor (IGF)-1, matrix metalloproteinase (MMP)-3 (R&D Systems, Minneapolis, MN, USA), MMP-2, MMP-9 (Uscn Life Science Inc., Hubei, PRC), MMP-13, angiostatin (Biosensis Pty Ltd, South Australia, Australia), and tissue inhibitors of metalloproteinase (TIMP)-1 and -2 (Raybiotec Inc., Norcross, GA, USA).

### Statistical analyses

Data are expressed as the mean ± standard deviation. Multiple comparisons were performed using analysis of variance with Dunnett’s test as appropriate. Differences were considered significant at *P* < 0.05.

## Results

### Cell markers of ASCs and ST2 cells

Cell markers of ASCs (passage two) and ST2 cells were identified using flow cytometry. ASCs expressed high levels of CD29, Sca-1, and CD90.2, whereas ST2 cells expressed high levels of CD29. Although both cell types expressed high levels of CD29, the markers CD34, CD117, Gr-1, CD11b, CD31, CD106, CD133, CD45, CD11c, and F4/80 were not detected in either cell type (Table 1).

**Table 1:** **Comparison of cell markers in ASCs and ST2 cells using flow cytometry**

**Marker**	**ASCs**	**ST2 cells**
CD29	95 ± 2	92 ± 2
Sca1	90 ± 1	74 ± 2
CD90.2	90 ± 1	5.3 ± 0.5
CD105	21 ± 1	3.7 ± 0.4
CD73	13 ± 2	7.5 ± 0.6
CD117	5.1 ± 0.8	11.5 ± 2
CD34	1 ± 1	0
CD133	1 ± 1	1 ± 1
CD45	0	0
Gr-1	0	0
CD11b	0	0
CD11c	0	0
F4/80	0	0
CD31	0	0

Percentage expressions of marker proteins are presented as means ± standard deviation. ASC, adipose tissue-derived stromal cell; ST2 cell, bone marrow-derived stromal cell.

### Migration of GFP^+^ bone marrow cells to tissue surrounding a wound, and to ASC and ST2 cell transplantation area

**Figure 1:**
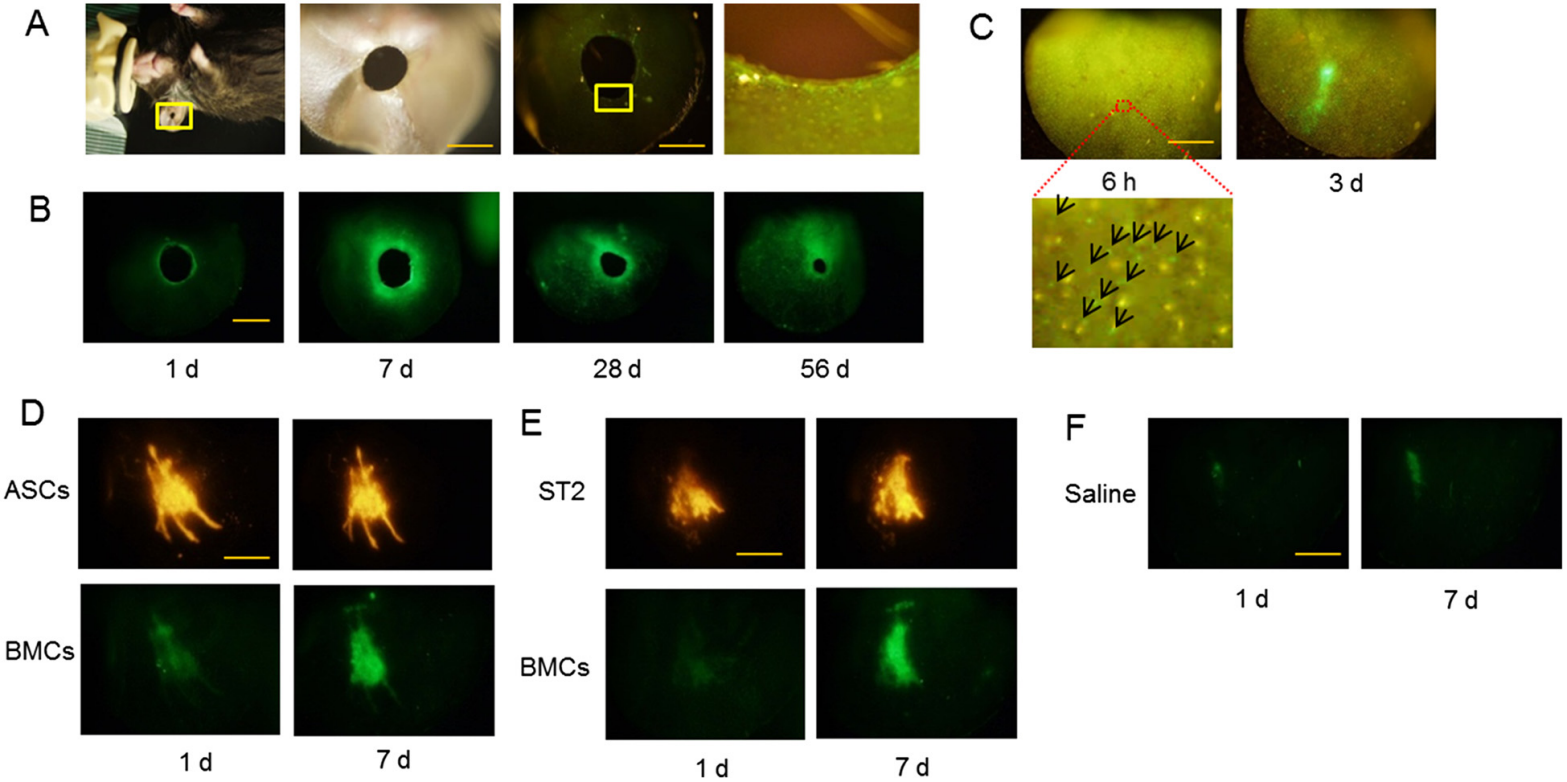
Observation of green fluorescent protein-positive (GFP^**+**^) bone marrow cells (BMCs) migrating to tissue surrounding a wound, and to adipose tissue-derived stromal cell (ASC) and bone marrow-derived stromal cell (ST2) transplantation area. **(A)** Bright field images of an ear in which was punched a hole (left two panels) and fluorescence images with a fluorescent filter for GFP (right two panels) at 5 hours after perforation. GFP^+^ BMCs migrated to tissue surrounding the hole (right panel). **(B)** Temporal changes in migration of GFP^+^ BMCs and sizes of holes. **(C)** Fluorescence images were taken using a fluorescent filter for GFP at 6 hours and at 3 days after intradermal transplantation of ASCs into mouse ears. GFP^+^ BMCs gradually migrated towards ASC transplantation area; arrows show the migrated GFP^+^ BMCs. **(D,E)** Fluorescence images of GFP^+^ BMC migration at 1 and 7 days after transplantation of PKH26 (red)-labeled ASCs **(D)** and PKH26-labeled ST2 cells **(E)**; images were taken using fluorescent filters for red fluorescent protein and GFP. **(F)** Fluorescence images at 1 and 7 days after saline injection. All scale bars = 2 mm.

Five hours after perforation of mouse ears with GFP^+^ BMC transplants, GFP^+^ cells migrated towards the hole (Figure 1A). Many GFP^+^ BMCs gradually migrated into the tissue around the hole (an additional movie file shows this in more detail, see Additional file 1), although the rate of migration decreased after peaking at 1 to 2 weeks, and the wound gradually closed (Figure 1B). The present mouse model was designed to identify BMCs that are related to wound healing. Accordingly, migration of GFP^+^ BMCs to the transplantation area was detected about 6 hours after transplantation with ASCs and ST2 cells, and accumulated in this area with time (Figure 1C-E; an additional movie file shows this in more detail, see Additional file 2). The migration of GFP^+^ BMCs toward transplantation area did not differ between ASCs and ST2 cells, and comparatively few BMCs migrated following injections of saline solution into ears (Figure 1F).

### Induction of inflammation following cell transplantation

**Figure 2:**
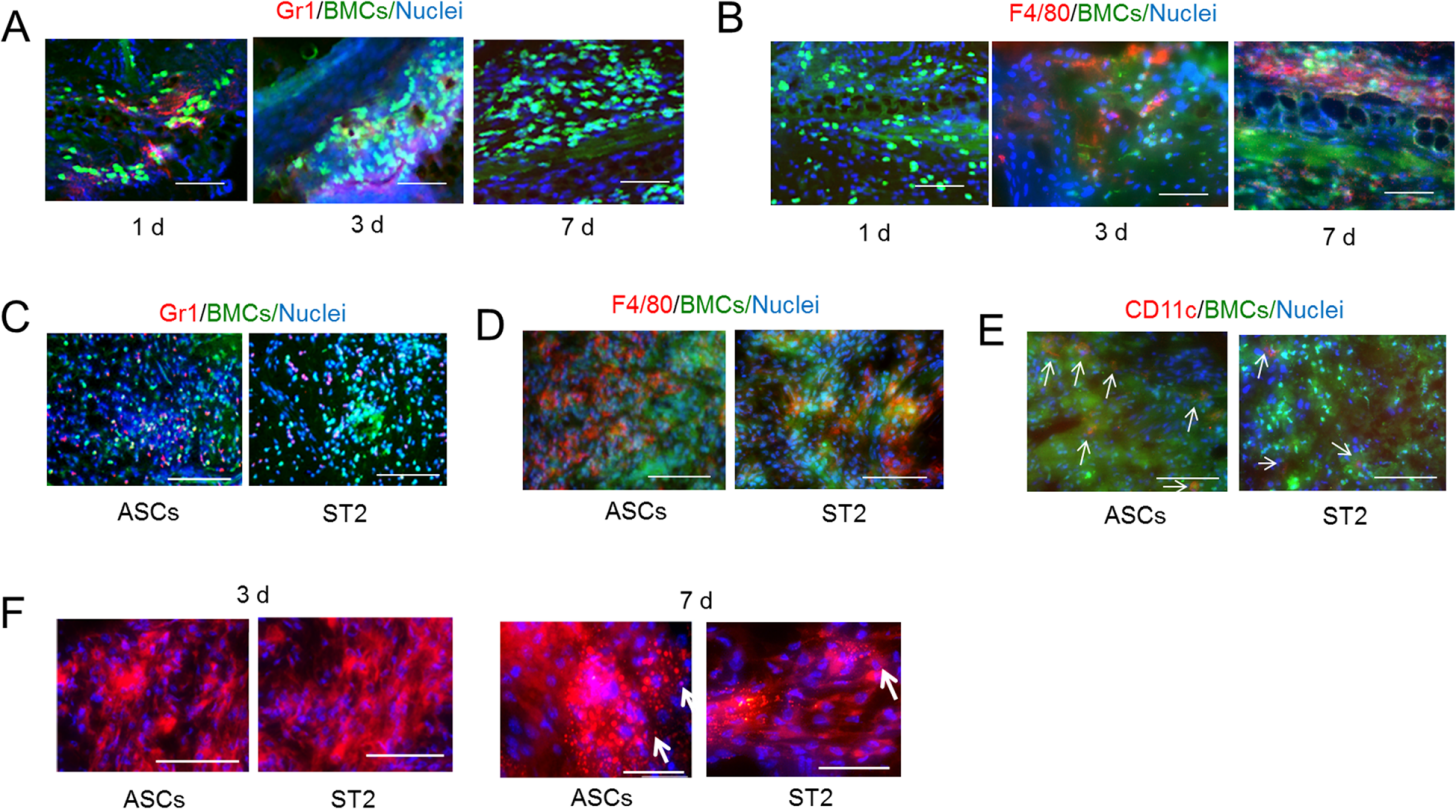
Identification of migrated cells, and morphological changes of transplanted adipose tissue-derived stromal cells (ASCs) and bone marrow-derived stromal cells (ST2). **(A)** Immunostaining of Gr-1 (red) at 1, 3, and 7 days after perforation and green fluorescent protein-positive (GFP^+^) bone marrow cell (BMC) migration (green). Scale bars = 50 μm. **(B)** Immunostaining of F4/80 (red) at 1, 3, and 7 days after perforation and GFP^+^ BMC migration (green). Scale bars = 50 μm. **(C)** Immunostaining of Gr-1 (red) at 1 day after transplantation of ASCs or ST2 cells and GFP^+^ BMC migration (green). Scale bars = 100 μm. **(D)** Immunostaining of F4/80 (red) at 7 days after transplantation of ASCs or ST2 cells and migration of GFP^+^ BMCs (green). Scale bars = 100 μm. **(E)** Immunostaining of CD11c (red) at 3 days after transplantation of ASCs or ST2 cells and GFP^+^ BMC migration (green). Scale bars = 100 μm. **(F)** Morphological changes of PKH26-labeled ASCs and ST2 cells (red) on days 3 and 7 after transplantation. Transplanted cells were engrafted after 3 days but were disrupted after 7 days. Arrows show lipids from PKH26 (lipophilic dye) linked membranes of burst cells. Scale bars = 50 μm. Nuclear staining was performed using Hoechst 33258 (blue).

One day after the holes were made in the ears, the migrated cells were predominantly Gr-1^+^. However, the presence of these cells peaked on day 3 and decreased to almost undetectable levels by day 7 (Figure 2A). In contrast, F4/80^+^ cells were not present on day 1 but were observed in the tissue surrounding the hole after day 3 (Figure 2B). On the day after ASC and ST2 cell transplantation, many migratory GFP^+^ cells were found to be positive for the granulocyte and neutrophil marker Gr-1 (Figure 2C) and both GFP-positive and -negative Gr-1^+^ cells were present. However, very few of these cells were present after 7 days. In contrast, F4/80^+^ cells (macrophages) were not observed on day 1 after cell transplantation but were present in the transplantation area after 7 days (Figure 2D) and included both GFP-positive and -negative cells. CD11c^+^ cells were detected on days 3 and 7 (Figure 2E) and their numbers marginally varied during the experiment. F4/80^+^ cell migration was more pronounced following transplantation of ASCs and ST2 cells than in tissue surrounding perforations. Although, both ASCs and ST2 cells were engrafted in the tissue at 3 days after transplantation, some transplanted cells had been disrupted after 7 days (Figure 2F).

### Inflammatory factors induced by interactions of ASCs or ST2 cells with inflammatory cells

**Figure 3:**
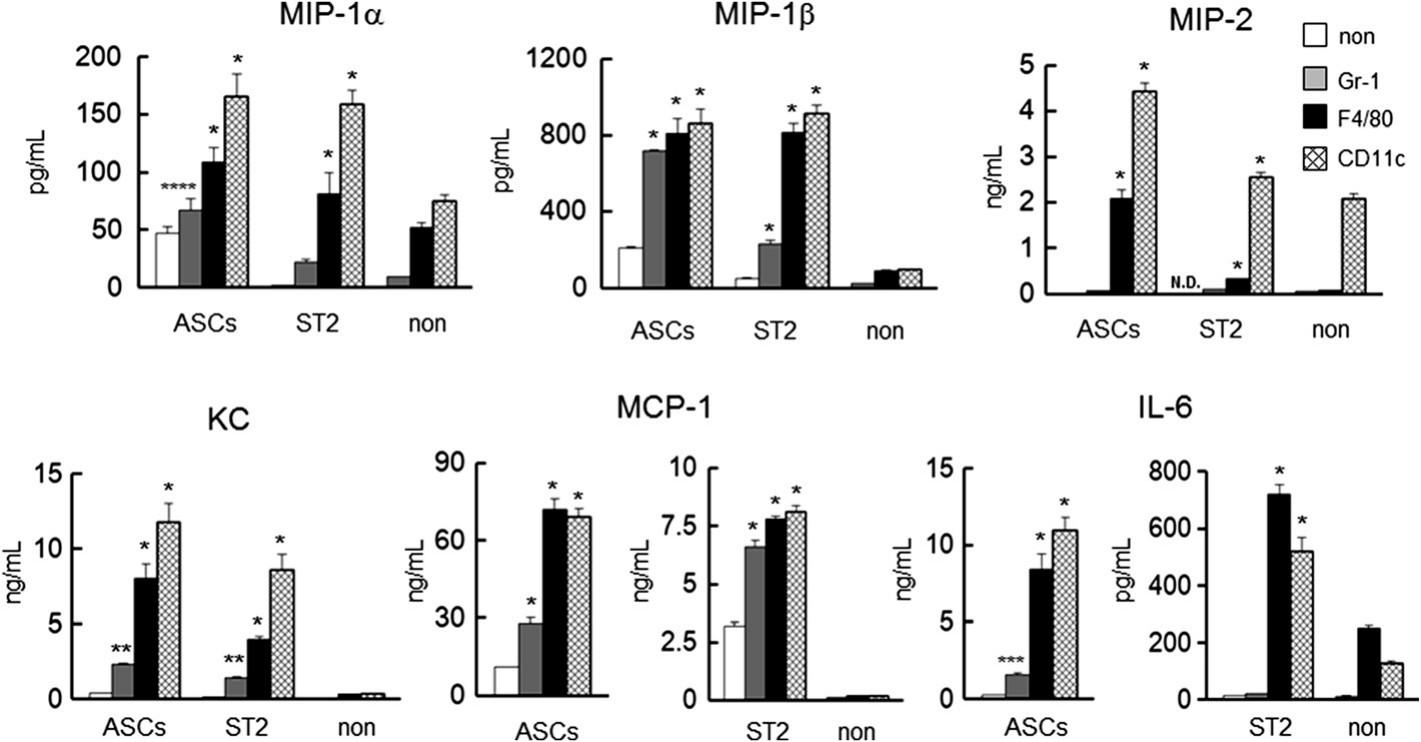
Secretions of the chemokines MIP-1α, MIP-1β, MIP-2, KC, and MCP-1 and the cytokine IL-6 following co-culture of ASCs or ST2 cells with Gr1^+^, F4/80^+^, or CD11c^+^ cells. Comparisons are made with monocultures (non). Data are presented as means ± standard deviation. *P < 0.0001, **P < 0.0005, ***P < 0.01, ****P < 0.05, versus monocultured cells; Dunnett’s test (*n* = 5). ASC, adipose tissue-derived stromal cell; IL, interleukin; KC, keratinocyte-derived chemokine; MCP, monocyte chemotactic protein; MIP, macrophage inflammatory protein; N.D., not detected; ST2 cell, bone marrow-derived stromal cell.

Inflammatory cells migrated toward the transplantation sites of both ASCs and ST2 cells (Figure 2). Hence cytokine and chemokine levels were determined in supernatants from cultured cells (Figure 3). In these experiments, ASCs secreted MIP-1α, MIP-1β, MIP-2, KC, MCP-1, and IL-6. However, differences between ASCs and ST2 cells were much greater than those anticipated from *in vivo* data (Figure 1C-E). Thus, ASCs or ST2 cells were co-cultured with Gr-1^+^, F4/80^+^, or CD11c^+^ cells. In these experiments, large quantities of secreted chemokines and cytokine were found in the media of monoculture of ASCs, and ASCs co-cultured with Gr-1^+^, CD11c^+^, or F4/80^+^ cells increased the secretions (Figure 3). Although monocultured ST2 cells had little secretory activity, the presence of Gr-1^+^, CD11c^+^, or F4/80^+^ cells promoted chemokine secretion from ST2 cells as well as ASCs. In particular, MIP-2 was absent in supernatants from monocultures of ST2 cells but was detected at levels similar to that in monocultures of ASCs after co-culture with inflammatory cells. Thus, paracrine secretions from both ASCs and ST2 cells were markedly increased in the presence of Gr-1^+^, F4/80^+^, or CD11c^+^ cells.

### Secretion of angiogenesis factors following interactions of ASCs or ST2 cells with inflammatory cells

**Figure 4:**
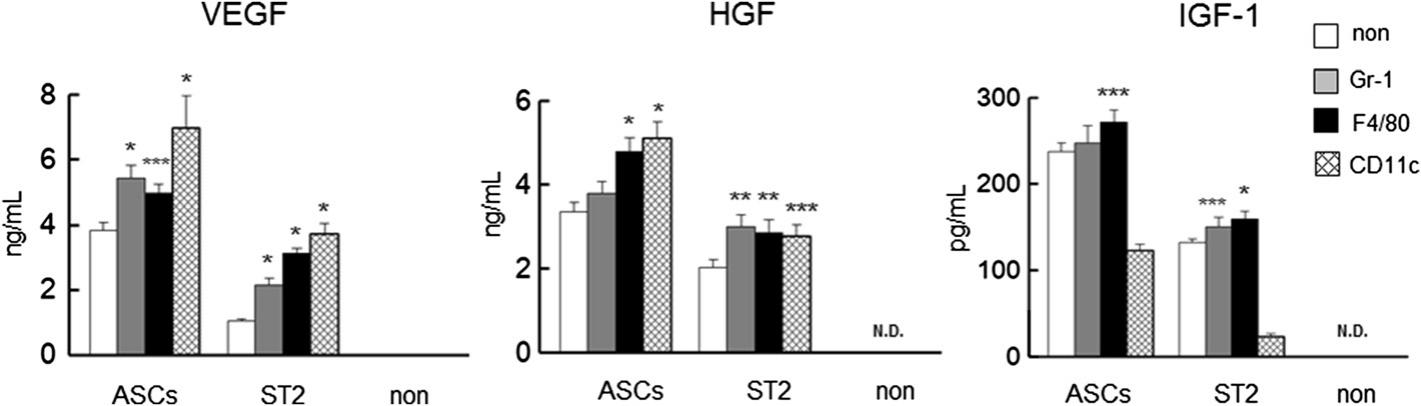
Secretions of the angiogenic factors VEGF, HGF, and IGF-1 following co-culture of ASCs or ST2 cells with Gr1^+^, F4/80^+^, or CD11c^+^ cells. Comparisons are made with monocultures (non). Data are presented as means ± standard deviation. *P < 0.0001, **P < 0.001, ***P < 0.005, versus monocultured cells; Dunnett’s test (*n* = 5). ASC, adipose tissue-derived stromal cell; HGF, hepatocyte growth factor; IGF, insulin-like growth factor; N.D., not detected; ST2 cell, bone marrow-derived stromal cell; VEGF, vascular endothelial growth factor.

Angiogenic growth factors such as VEGF, HGF, and IGF-1 were measured in monoculture supernatants and in media from co-cultures of ASCs or ST2 cells with Gr-1^+^, F4/80^+^, or CD11c^+^ cells (Figure 4). ASCs abundantly secreted angiogenic growth factors alone and in co-culture with inflammatory cells. However, in comparison with monocultured ST2 cells, co-culture ST2 cells with Gr-1^+^, F4/80^+^ or CD11c^+^ cells increased the secretions of VEGF and HGF. The decreased secretion of IGF-1 was observed in co-culture of ASCs or ST2 cells with CD11c^+^ cells.

### Secretion of matrix metalloproteinases following interactions of ASCs or ST2 cells with inflammatory cells

**Figure 5:**
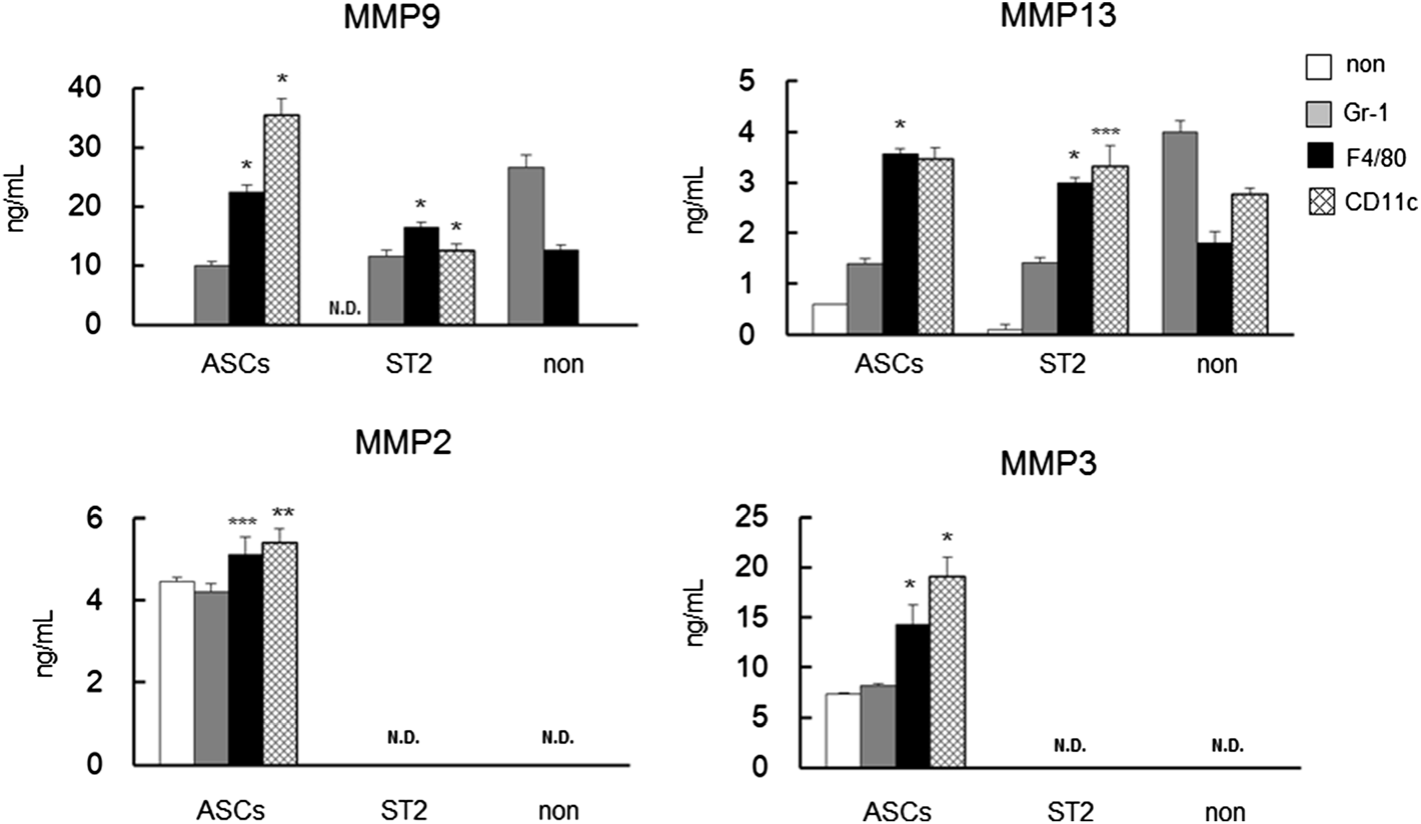
Secretions of MMP-2, -3, -9, and -13, following co-culture of ASCs or ST2 cells with Gr1^+^, F4/80^+^, or CD11c^+^ cells. Comparisons are made with monocultures (non). Data are presented as means ± standard deviation. *P < 0.0001, **P < 0.0005, ***P < 0.01, versus monocultured cells; Dunnett’s test (*n* = 5). ASC, adipose tissue-derived stromal cell; MMP, matrox mettaloproteinase; N.D., not detected; ST2 cell, bone marrow-derived stromal cell.

MMP-2, -3, -9, and -13 secretions were determined in media from co-cultures of ASCs or ST2 cells with Gr-1^+^, F4/80^+^, or CD11c^+^ cells (Figure 5). Compared with monocultured ASCs and ST2 cells, the secretions of MMP-9, and -13 were markedly increased in the presence of F4/80^+^ and CD11c^+^ cells. However, monocultured Gr-1^+^ cells secreted large quantities of MMP-9 and MMP-13, and the presence of these proteins was decreased in media from co-cultures with ASCs or ST2 cells. In contrast, MMP-2 and -3 were detected at high levels in supernatants from both mono- and co-cultured ASCs but were undetectable in all ST2-containing cell cultures.

### Secretion of anti-inflammatory factors, anti-angiogenesis factors, and matrix metalloproteinase inhibitors following interactions of ASCs or ST2 cells with inflammatory cells

**Figure 6:**
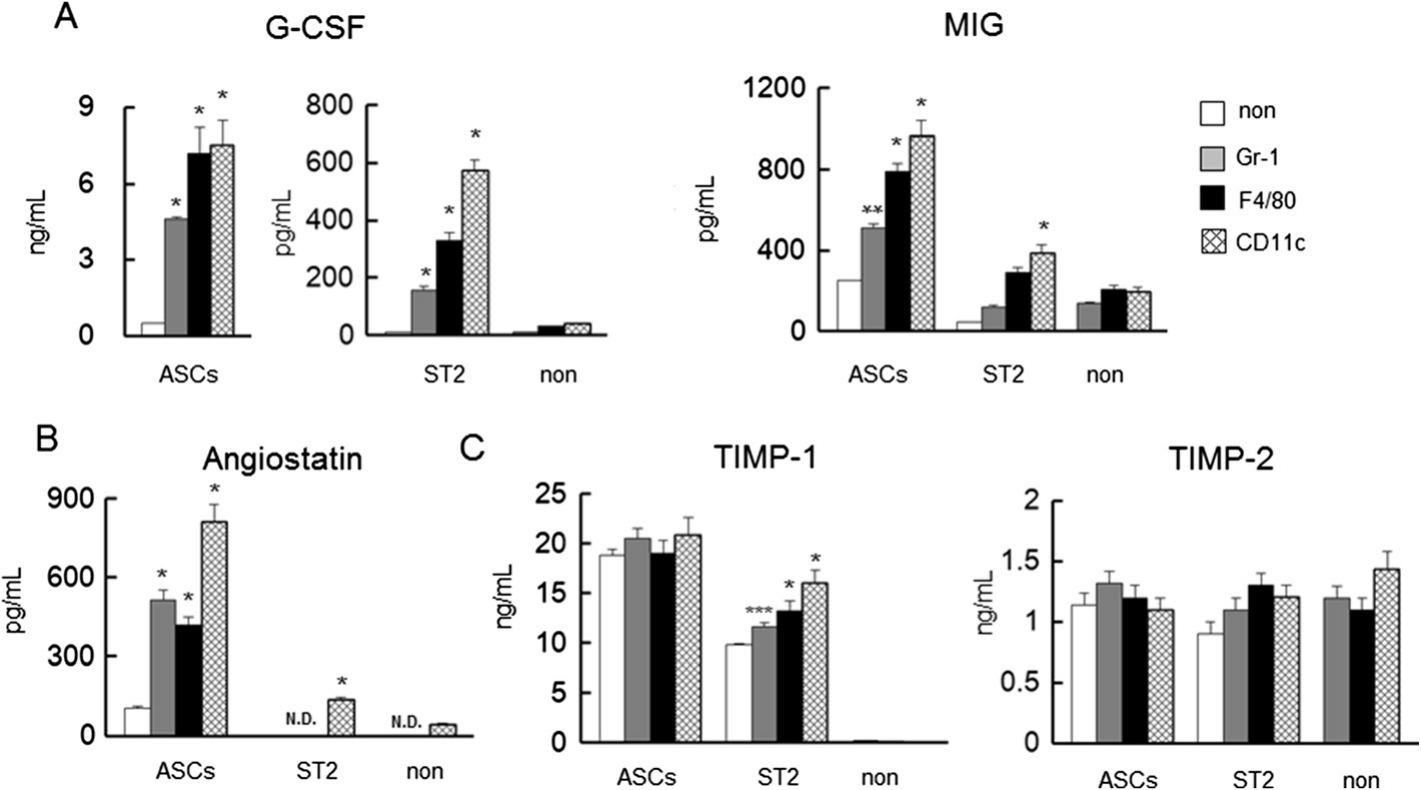
Secretions of the anti-inflammatory and the anti-angiogenic factors G-CSF, MIG, and angiostatin, and TIMP-1 and TIMP-2, following co-culture of ASCs or ST2 cells with Gr1^+^, F4/80^+^, or CD11c^+^ cells. **(A)** Secretions of G-CSF and MIG. **(B)** Secretion of angiostatin. **(C)** Secretions of TIMP-1 and TIMP-2. Comparisons are made with monocultures (non). Data are presented as means ± standard deviation. *P < 0.0001, **P < 0.0005, ***P < 0.05, versus monocultured cells; Dunnett’s test (*n* = 5). ASC, adipose tissue-derived stromal cell; G-CSF, granulocyte-colony stimulating factor; MIG, monokine induced by interferon γ; N.D., not detected; ST2 cell, bone marrow-derived stromal cell; TIMP, tissue inhibitors of metalloproteinase.

Monocultured ASCs secreted inflammatory factors, angiogenesis factors, and MMPs at high levels, whereas ST2 cells secreted these proteins only in the presence of inflammatory cells. In contrast, anti-inflammatory G-CSF secretions from both cell types were dramatically increased in co-cultures with inflammatory cells (Figure 6A). Moreover, MIG secretions from both ASCs and ST2 cells increased by 2- to 2.5-fold in co-culture with CD11c^+^ cells (Figure 6A). Among inflammatory cells, only CD11c^+^ cells secreted the anti-angiogenic factor angiostatin. Both mono- and co-cultured ASCs secreted angiostatin, whereas ST2 cells only secreted angiostatin in the presence of CD11c^+^ cells (Figure 6B). Co-culture with inflammatory cells did not affect the secretion of TIMP-1 from ASCs or the secretion of TIMP-2 from both ASCs and ST2 cells (Figure 6C).

## Discussion

Induction of inflammatory factors as well as accumulation of various types of inflammatory cells is critical to self-regeneration during wound healing of animal organs [6]. Accordingly, inflammatory cells and secretory factors initiate tissue regeneration by replenishing cells and extracellular components. In the present *in vivo* imaging experiments, bone marrow-derived inflammatory cells were involved in the first stage of the wound healing process in the punched hole of the mouse ears. Moreover, in subsequent ASC and ST2 cell culture experiments, the expression of cell markers differed between cell types (Table 1) and as ASCs secreted high levels of angiogenic growth factors, cytokines, and chemokines *in vitro*, we expected more extensive angiogenic effects with ASCs than with ST2 cells *in vivo*. However, *in vivo* observations of angiogenesis and inflammation showed no significant differences between the effects of transplanted ASCs and ST2 cells, although ASCs produced greater quantities of angiogenic growth factors such as VEGF, HGF, and IGF-1 than ST2 cells. In contrast, co-culture of ST2 cells with inflammatory Gr1^+^ cells (neutrophils, granulocytes) or F4/80^+^ cells (macrophages) markedly increased the production of angiogenic growth factors. These data suggest that ST2 cells acquire angiogenic potential following interactions with inflammatory cells, which may migrate toward chemotactic inflammatory factors such as MIP, KC, MCP-1, and IL-6 [11,12]. Moreover, following exposure to inflammatory cells, these proteins were secreted at high levels from both cell types, indicating that ASCs and ST2 cells exert similar effects under these conditions. In further experiments, extensive secretion of chemokines and growth factors from ASCs was associated with hemangioma formation, with hyper-inflammation and extensive angiogenesis. However, in co-culture with inflammatory cells, ASCs secreted high levels of growth factors, anti-inflammatory factors, and angiogenic inhibitors such as G-CSF [13], MIG [14], angiostatin [15], and IL-6, which act as both pro-inflammatory and anti-inflammatory factors [16,17]. In contrast, secretions of pro-inflammatory and angiogenic factors were less abundant from ST2 cells than from ASCs and did not include anti-inflammatory factors or inhibitors of angiogenesis. Thus, inflammatory and angiogenic processes are transient in healthy organisms, and a dynamic balance of pro- and anti-inflammatory, and pro- and anti-angiogenic factors, MMPs, and TIMPs, act as accelerators and brakes, respectively. Accordingly, transplanted ASCs and ST2 cells were eradicated in healthy mice (Figure 2D,E), reflecting phagocytosis by macrophages and a healthy balance of inflammatory processes.

Macrophages are classified as pro-inflammatory (classically activated M1 macrophage phenotype) or anti-inflammatory (alternatively activated M2 macrophage phenotype) [18]. In the present studies using CD11c as a marker of pro-inflammatory macrophages [19], few CD11c^+^ cells had migrated into the ASC and ST2 cell transplantation area at 3 days after the transplantation, although migrant F4/80^+^ cells were abundant at 7 days. Moreover, the migration of pro-inflammatory macrophages was greater after transplantation of ASCs and ST2 cells than after perforation of ear tissues. However, disruption of transplanted ASCs and ST2 cells was observed after 7 days and not all transplanted cells become engrafted, indicating that stromal cell transplants act as initial inducers of inflammation in the first stage of the wound healing process. Subsequently, these cells are attacked by neutrophils, granulocytes, and pro-inflammatory macrophages and are then phagocytosed by macrophages that mediate paracrine effects and facilitate the recovery of damaged tissue in a transplant-dependent fashion.

Cell-based therapies using ASCs are highly efficacious for decubitus ulcers [20,21], reportedly reflecting the actions of angiogenenic factors, such as HGF and VEGF [22]. Among MMPs that are involved in cutaneous wound healing [23], MMP-9 plays a role in keratinocyte migration and epithelialization, whereas MMP-13 plays a role in epithelialization, angiogenesis, and contraction of wound healing processes [24]. In the present study, the presence of ASCs or ST2 cells inhibited excessive secretion of MMP-9 and MMP-13 from neutrophils but caused excessive secretion of MMP-9 and MMP-13 in the presence of macrophages (Figure 5). Thus, in addition to angiogenic factors, MMP-9 and MMP-13 may play a role in wound healing in patients with diabetes. However, previous studies suggest that high expression of MMP-9 results in inadequate wound healing of foot ulcers in patients with diabetes [25] and contribute to exercise inflammation. High expression of MMP-9 is reportedly associated with protein tyrosine phosphatase-1B in skin, and aberrant serum growth factor levels prevent healing of diabetic foot ulcers [26]. In another report, because the ratio of MMP-9 and TIMP-1 is positively correlated with poor healing foot ulcers in patients with diabetes [27], we surmise that the issue is not high expression of MMP-9 but an inadequate ratio of MMP-9 and TIMP-1.

Both of the present cell types have been investigated as cell-based therapies for autoimmune diseases such as rheumatoid arthritis and osteoarthritis [28-30]. Moreover, ASCs and BMCs have potential hematopoietic roles in osteogenesis, chondrogenesis, adipogenesis, myogenesis, and angiogenesis [31-33], and previous comparisons of ASCs and BMCs show similar potential properties *in vivo* and *in vitro* [25,34-38]. ASC transplantations have also been effective in *in vivo* experiments, which showed that ASCs induce immune tolerance and prevent arthritis by secreting both pro-inflammatory and anti-inflammatory factors [39,40]. Moreover, articular rheumatism induces intense synovial inflammation and pannus formation due to excessive synoviocyte growth, leading to erosion of bones and disruption of cartilage tissues. Joint tissue comprises various cell types, such as fibroblasts, endothelial cells, and inflammatory cells, which further comprise neutrophils, macrophages, and T cells, which secrete various proteases and cytokines. Accordingly, MMPs secreted from chondrocytes and activated synoviocytes are involved in cartilage destruction [41], and MMP-3 and IL-6 are known markers of rheumatoid arthritis and osteoarthritis. The present data show markedly elevated secretion of MMPs in co-cultures of ASCs with inflammatory cells, particularly with macrophages (Figure 5), although TIMP-2 secretions were not affected by co-culture with inflammatory cells (Figure 6C). In contrast, ST2 cells did not secrete MMP-2 or MMP-3, and their interactions with inflammatory cells were weaker than those of ASCs, suggesting that secretions of various MMPs and chemokines and stimulation of inflammatory cells may complicate the use of ASCs for the treatment of rheumatoid arthritis or osteoarthritis. In particular, ASCs may replace bone marrow with adipose tissues and cause bone loss in the elderly [42]. Accordingly, a close relationship has been shown between osteoblasts and adipocytes [43], which moderate each other inversely [44]. Hence, prolonged inflammation may ultimately cause or exacerbate chronic inflammatory disease.

## Conclusions

Transplantation of ASCs and ST2 cells induced the migration of bone marrow-derived inflammatory cells such as neutrophils and macrophages. Thus, the effects of cell-based therapy using ASCs and ST2 cells are depended on paracrine effects that are mediated by chemokines, cytokines, growth factors, and MMPs, which are expressed in response to interactions between transplanted cells and bone marrow-derived inflammatory cells. Accordingly, the present data show that the paracrine effects of transplanted cells are influenced by inflammatory cells and maintained by a balance of secreted inhibitors. Thus, the use of ASCs for cell-based therapy requires close attention in patients with a history of chronic inflammatory disease.

## Additional files

Additional file 1:
**Real-time image of the 7th day after making a hole to the ear of the mouse into which GFP** ^**+**^ **BMCs were injected.** GFP^+^ BMCs circulating in vessels migrated into the surrounding tissue of a 2-mm diameter hole. Additional file 2:
**Real-time image of transplanting PKH26-labeled ASCs into the ear of the mouse in which GFP** ^**+**^ **BMCs were injected.** The first image is GFP^+^ BMCs circulating in vessels immediately after injection, and the second is immediately after PKH26-labeled ASCs were transplanted. The next one is GFP^+^ BMCs migrating into the transplantation site of PKH26-labeled ASCs on the 7th day after the transplantation, and the last one is the magnified image.

## Abbreviations

ASC: adipose tissue-derived stromal cell
BMC: bone marrow cell
DMEM: Dulbecco’s modified Eagle’s medium
G-CSF: granulocyte-colony stimulating factor
GFP: green fluorescent protein
HGF: hepatocyte growth factor
IGF: insulin-like growth factor
IL: interleukin
KC: keratinocyte-derived chemokine
MCP: monocyte chemotactic protein
MIG: monokine induced by interferon γ
MIP: macrophage inflammatory protein
MMP: matrix metalloproteinase
PBS: phosphate-buffered saline
ST2 cell: bone marrow-derived stromal cell
TIMP: tissue inhibitors of metalloproteinase
VEGF: vascular endothelial growth factor
